# Potential Drug-drug Interaction among the Patients Admitted in Intensive Care Units of a Tertiary Care Centre: A Descriptive Cross-sectional Study

**DOI:** 10.31729/jnma.7137

**Published:** 2022-03-31

**Authors:** Rakesh Ghimire, Pravin Prasad, Subigya Parajuli, Rabin Basnet, Pratik Lamichhane, Nirmal Poudel, Pramesh Sunder Shrestha, Shristi Kharel, Akritee Pokharel, Anish Mudvari

**Affiliations:** 1 Department of Clinical Pharmacology, Maharajgunj Medical Campus, Institute of Medicine, Maharajgunj, Kathmandu, Nepal; 2 Manmohan Cardiothoracic Vascular and Transplant Center, Maharajgunj, Kathmandu, Nepal; 3 Department of Anesthesiology, Tribhuvan University Teaching Hospital, Institute of Medicine, Maharajgunj, Kathmandu, Nepal; 4 Maharajgunj Medical Campus, Institute of Medicine, Maharajgunj, Kathmandu, Nepal; 5 International Friendship Children's Hospital, Maharajgunj, Kathmandu, Nepal

**Keywords:** *drug interactions*, *intensive care units*, *Nepal*, *software*

## Abstract

**Introduction:**

Drug interactions are one of the major contributors to increase hospital stay, inflate health care expenses, and cause serious adverse events and end-organ damage. Patients admitted to the intensive care unit are already critically sick and are at greater risk of these adverse outcomes. The study aimed to find out the prevalence of potential drug-drug interactions in the intensive care units of a tertiary care centre.

**Methods:**

A descriptive cross-sectional study was conducted among the patients admitted in the intensive care unit of a tertiary care hospital from April-June 2019. Ethical approval was taken from the Institutional Review Board at the institute (Reference number: 399). Convenience sampling method was used. Data was collected using proforma and potential drug-drug interactions were identified using Lexicomp® drug-interactions version 1.1 (Wolters Kluwer). All the drug interactions identified were classified and the severity scale of interactions was also defined. Statistical Package for the Social Sciences version 17.0 was used for data analysis. Point estimate at 95% Confidence Interval was calculated along with frequency, percentage, mean, standard deviation, and mode.

**Results:**

Out of 101 patients, the prevalence of the drug-drug interaction was found to be 90 (89.11%) (83.04-95.18 at 95% Confidence Interval). A total of 490 drug-drug interactions were identified. In severity scale, it was seen that 311 (63.46%) were of moderate severity and 303 (61.83%) of drug interactions were categorised as category C in risk rating.

**Conclusions:**

Prevalence of potential drug-drug interactions was higher compared to similar published literature. The most common drug with potential interaction was fentanyl and among pairs was fentanyl plus paracetamol.

## INTRODUCTION

Alteration in the efficacy or toxicity of one drug due to the presence of another simultaneously administered drug is termed as drug-drug interactions (DDI).^1^ The prevalence of DDI is reported to be 3-5% in patients who receive 3-10 drugs and increases to 20% when they are given 10-20 drugs.^2^ Factors like polypharmacy (four or more drugs), pharmacological properties of medicines, elderly patients having comorbidities predisposed to the development of DDIs.^3-5^ DDIs are known to increase hospital stay, inflate health care expenses, and cause serious adverse events.

Most patients admitted to the Intensive Care Units (ICU) have multiple co-morbidities and are being managed with polypharmacy. So, there is always a substantial risk of potential drug-drug interaction (pDDI) in the ICU setting. Data from our own country shows that 57.7% of ICU admitted patients experienced at least one DDI in one of the teaching hospitals in Nepal.^6^

The aim of this study was to find out the prevalence of potential DDIs in ICU patients of a tertiary care centre.

## METHODS

This was a descriptive cross-sectional study conducted among the patients admitted in the ICU of Tribhuvan University Teaching Hospital, Institute of Medicine. Ethical approval was taken from the Institutional Review Committee of Institute of Medicine (Reference number: 399). All the patients who were prescribed more than two drugs admitted in the ICU for more than 24 hours between April-June 2019 were included in the study. Consent was obtained from the patient's relative who had consented during hospital admission. However, patients admitted in ICU for less than 24 hours, receiving topical drugs (ointments, creams, eye drops, or ear drops), nutritional supplements, and vitamins only, and those patients whose prescription was less than two drugs were excluded. A convenience sampling method was used.

The sample size was calculated using the following formula:

n = (Z^2^ × p × q) / e^2^

  = (1.96^2^ × 0.5 × 0.5) / 0.1^2^

  = 97

Where,

n = minimum required sample size
Z = 1.96 at 95% Confidence Interval (CI)
p = prevalence of pDDI taken as 50% for maximum sample size
q = 1-p
e = margin of error, 10%

Patients' cardex were reviewed on a daily basis till the discharge from ICU and data was collected in the proforma sheet that included demographic data of patient and the drugs given to the patient. The enlisted drugs were assessed for pDDIs which was identified by the Lexicomp® drug-interactions version 1.1 (Wolters Kluwer). Their risk rating was classified into five categories.^7^ The identified pDDIs were also classified according to the severity.^6^

Collected data was coded and entered in Microsoft Excel and was analysed using Statistical Package for the Social Sciences (SPSS) version 17.0. Point estimate at 95% Confidence Interval was calculated along with frequency, percentage, mean, standard deviation and mode.

## RESULTS

Out of 101 patients admitted to ICU, the prevalence of potential drug-drug interaction was found in 90 (89.11%) patients (83.04-95.18 at 95% Confidence Interval). The total number of drugs prescribed was 816 and the mean number of drugs prescribed per prescription was found to be 8.08±3.7 (range 3-25).

Fentanyl was found to be the most common drug accounted for 238 (29.28%) followed by phenytoin which was 124 (25.30%) responsible for pDDIs. When drugs were classified according to Anatomical and Therapeutic Category (ATC), it was seen that nervous system drugs (ATC class N) were the most common drugs which was 501 (51.1%) among the observed pDDIs, followed by anti-infective drugs (ATC class J) 141 (14.4%).

The mean number of pDDI per prescription was found to be 4.85±4.56. In 36 (40%) patients, there were more than five pDDIs. A total number of 490 pDDIs were identified. On severity rating, it was seen that 311 (63.46%) pDDIs identified were of moderate severity. On categorising the pDDIs according to risk rating, it was seen that 303 (61.83%) fell into category C. The pDDIs that were classified as category X was found to be 10 (2.04%) (Table 1).

**Table 1:** Distribution of identified pDDIs according to their severity rating and risk rating (n = 490).

Description	n (%)
**Severity rating of pDDIs**	
Minor	86 (17.55)
Moderate	311 (63.46)
Major	93 (18.97)
**Risk rating of pDDIs**	
A	4 (0.81)
B	82 (16.73)
C	303 (61.83)
D	91 (18.57)
X	10 (2.04)

Among categoryX pDDIs, quetiapine andmetoclopramide drug pairs were most common foundin 3 (30%) (Table 2).

**Table 2:** Drug pairs that were found to have category X pDDIs in risk rating (n = 10).

Drug 1	Drug 2	n (%)
Quetiapine	Metoclopramide	3 (30)
	Potassium chloride	2 (20)
	Amiodarone	1 (10)
Nimodipine	Phenytoin	1 (10)
Morphine	Linezolid	1 (10)
Chlorpromazine	Metoclopramide	1 (10)
Atorvastatin	Voriconazole	1 (10)

**Figure 1:**
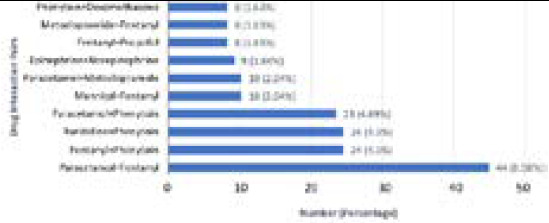
The drug pairs with the ten most common pDDIs with their frequency.

The most common drug pair causing pDDI was paracetamol and fentanyl 44 (8.98%) with risk rating B and minor severity. The second most common drug pair causing pDDI was fentanyl and phenytoin and fell under risk rating C with moderate severity (Figure 1).

## DISCUSSION

This study demonstrated a prevalence of 89.11% of pDDI. Similar prevalence of pDDI was reported by a study conducted in ICUs of public and private hospitals in Brazil.^8^ However, the study conducted by another study in a public hospital in Brazil reported that 22% of ICU prescriptions studied had 787 identified DDIs.^9^ Another study from India conducted in geriatric patients admitted in ICU reported prevalence of pDDI to be 83.25%.' Studies with lower rates (72.5%) of prevalence of pDDIs are also present.^10^ A study from a medical college of Pokhara, Nepal reported 15 out of 26 patients admitted in their ICU to have encountered at least one pDDI.^6^

Several factors could have contributed to the high prevalence of pDDI seen in our study. The factors could be drug related or patient related. Patients in ICU are critically ill and have derangements in their physiological state due to multiple health issues. This leads to complex pharmacotherapy being instituted in them that involves use of multiple drugs (polypharmacy). Thus, these patients are predisposed to witness higher pDDIs as compared to patients in other settings. The prevalence of pDDIs in this study was significantly correlated with the numbers of drugs administered. A study reported that frequency of pDDIs were significantly correlated with the number of medications prescribed. Additionally, the study also reported correlation of pDDI with factors like age and number of co-morbidities of the patient.^1^ A study from Brazil has reported that the number of DDI is positively correlated with duration of hospital stay, irrespective of the number of prescribed drugs and procedures performed in the patients from ICU of a general hospital.^9^

The potential drug-drug interactions detected were mostly moderate to severe. The clinical evidence of a drug interaction is determined by the severity.^11,12^ The pDDIs encountered in a study conducted in Nepal reported 72.23% of pDDIs as of moderate severity according to the Micromedex electronic database system.^6^ A study from Brazil had reported 27.13% of pDDIs to be highly significant, ones with either severe or moderate intensity and with either established or probable evidence.^8^ Though there were smaller number of minor pDDIs seen in our study, the interactions could be clinically relevant due to the clinical condition of the ICU patients as suggested by other authors.^8^ The higher prevalence of moderate pDDIs seen in our study is concerning and needs to be addressed. Several interventions like Continuing Medical Education targeting physicians and nurses, drug bulletins, audits, etc. could help to create awareness among the physicians to avoid them in future. The involvement of expertise like Clinical Pharmacologists in the team to share the responsibility of patient care could also help to minimise pDDIs. Availability of a reliable drug interaction checker software at the point of care could also help minimise the prevalence of pDDIs.

When the drugs implicated to cause pDDIs were classified according to ATC classification, it was seen that the drugs acting on the nervous system were most commonly associated with pDDIs (51.1%). Fentanyl was the most common drug with a high probability of causing pDDI in our study. A Brazilian study similarly reported 40% of pDDIs being associated with drugs acting in the central nervous system and also reported midazolam (20.8%) to be the most common drug associated with pDDIs followed by fentanyl (6.7%) and both drugs were combinedly associated with 45 (14.5%) of pDDIs identified in the study.^10^ Pain is a common complaint of the patients admitted in ICU which could be due to the morbidity as well as iatrogenic (associated with the procedures). It is recommended that procedural pain in adult ICU patients is managed appropriately using opioids like fentanyl along with other pharmacological and non-pharmacological modalities.^13^

Fentanyl-paracetamol pair was the most common drug pair causing DDI followed by fentanyl-phenytoin pair. According to the output of software used to check the potential pDDIs in this study, fentanyl may decrease the absorption of paracetamol. However, the extent to which the systemic exposure of paracetamol is decreased is uncertain. The severity rating of this potential pDDIs is minor and the risk rating is B. Phenytoin is an enzyme inducer and increases the activity of CYP4503A4, an enzyme involved in metabolising fentanyl. Thus, a patient receiving these agents concomitantly might experience reduced efficacy of fentanyl or may experience withdrawal symptoms. Different studies have reported different drug-drug pairs as the most common contributor of pDDI. A study from Nepal conducted in ICU patients identified aspirin-angiotensin-converting enzyme (ACE) inhibitors as the most common drug interaction pair in their study.^6^ Another study conducted in 2008 reported that captopril-spironolactone pair was the most frequent (27 prescriptions) implicated to cause highly significant pDDIs in their ICU patients.^8^ In another study from Brazil, it was seen that there were 64 distinct pair of drugs causing DDIs and among them insulin-aspirin pair was the most commonly implicated to cause DDIs (106 pDDIs) in the ICU patients at that general hospital.^9^ The difference in pattern of pDDIs could have been due to variation in admission diagnosis, availability of the drugs, and the P-drug of the treating physicians. There were seven drug pairs in our study contributing to ten pDDIs (2%) in our study that was categorised as "X" in risk rating. Similar proportion of category X pDDI was reported by Shetty V, et.al. who found 14 drug pairs contributing to 20 DDIs (3.02%) belonging to category X.^1^ In our study, quetiapine-metoclopramide pair was the most common cause of category X pDDIs. Both the drugs are known to increase antidopaminergic effects and may lead to development of extrapyramidal effects and neuroleptic malignant syndrome.

This was a descriptive cross-sectional study done in a single tertiary care centre. In the current study, though, occurrence of pDDIs was not monitored clinically. Duration of hospital stay was also not calculated. The small-time frame also excluded the chances of patients with large varieties of comorbidities to be included. The software used for identification of drug interaction has sensitivity of 97% and specificity of 90%. The positive and negative predictive values of the software are similarly 90% and 97% respectively. The software additionally does not take into consideration the dose of the medicines while assessing the drug-pairs for drug interactions.^14^

## CONCLUSIONS

The prevalence of potential drug-drug interactions was high as compared to findings from similar reported literature. The potential drug interactions identified were of moderate severity. The most common drug to have potential drug interaction was fentanyl and the medication pair was fentanyl plus paracetamol. A small percentage of drug interactions belonged to the risk category X. The outcomes of this study will aid in raising awareness of pDDIs in ICU.
